# National Hospital for the Paralysed and Epileptic

**Published:** 1902-01-04

**Authors:** B. Burford Rawlings

**Affiliations:** Secretary and Director


### Editor's Letter-Box.

[Our Correspondents are reminded that prolixity is a great bar to publication, and that brevity of style and conciseness of statement greatly facilitate early insertion.]

### NATIONAL HOSPITAL FOR THE PARALYSED AND EPILEPTIC.

*To the Editor of* THE HOSPITAL.

SIR,—In your summarised report of the speeches at the meeting on the 12th inst., a prominent speaker is made to say—"it was very sad that he (Mr. Rawlings) should have to be removed."

For reasons well understood I was not present at the meeting, but I think your reporter must be in error.

I shall be obliged if you will allow me to say that immediately upon the appearance of Sir Edward Fry's report I announced my intention to retire, and I never for a moment entertained the idea of accepting any office alternative to that which, pending the completion of arrangements, I have continued to hold at the wish of all parties.

I am, sir, your obedient servant,

B. BURFORD RAWLINGS,

December 24th, 1901. Secretary and Director.

